# Supplementary material for: Single-Cell Analysis Differentiates the Effects of p53 Mutation and p53 Loss on Cell Compositions of Oncogenic Kras-Driven Pancreatic Cancer
Source: Cells. 2023 Nov 12;12(22):2614. doi: 10.3390/cells12222614 (PMC10670612; doi:10.3390/cells12222614)

Supplementary Figure S1

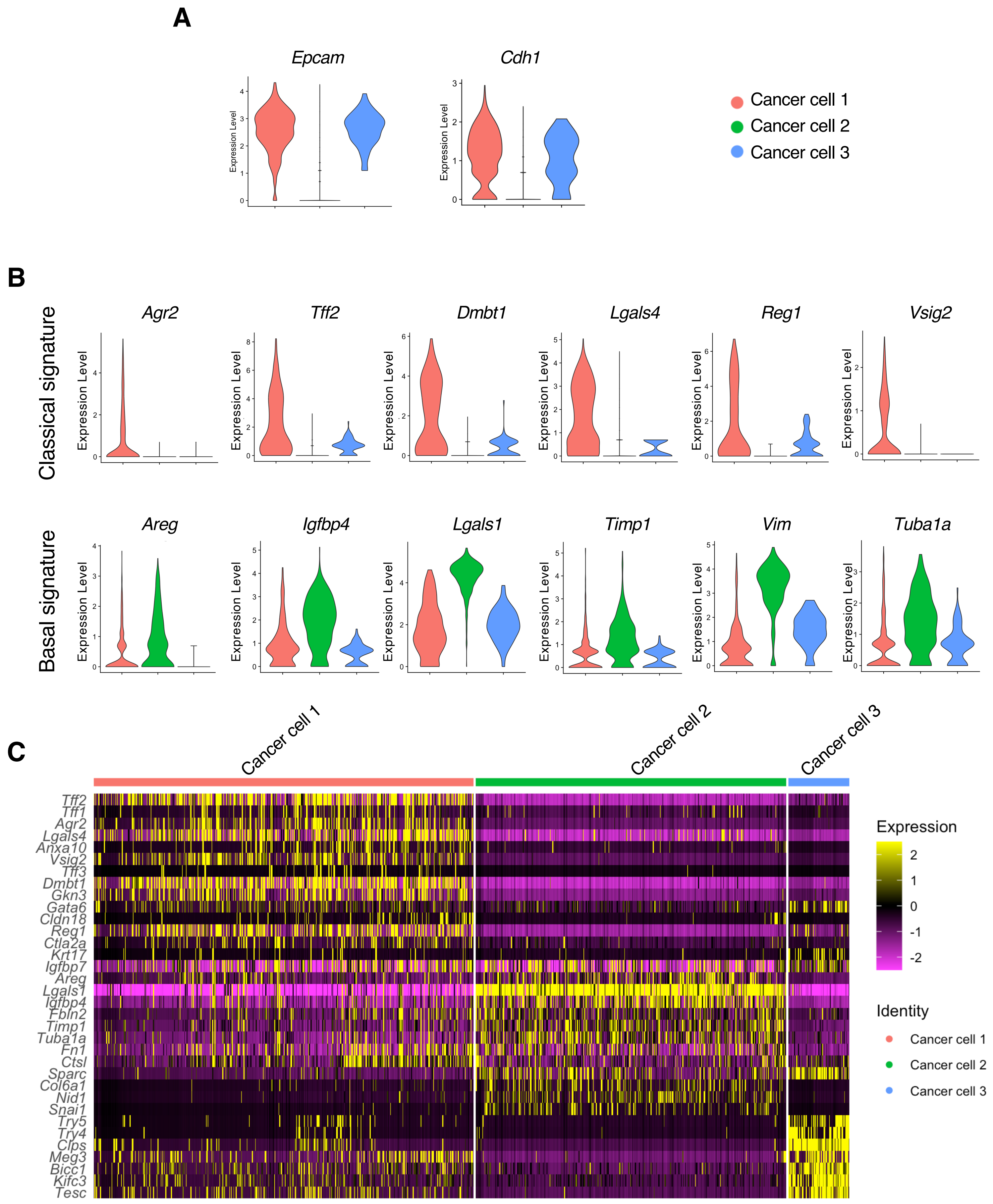

Supplementary Figure S2

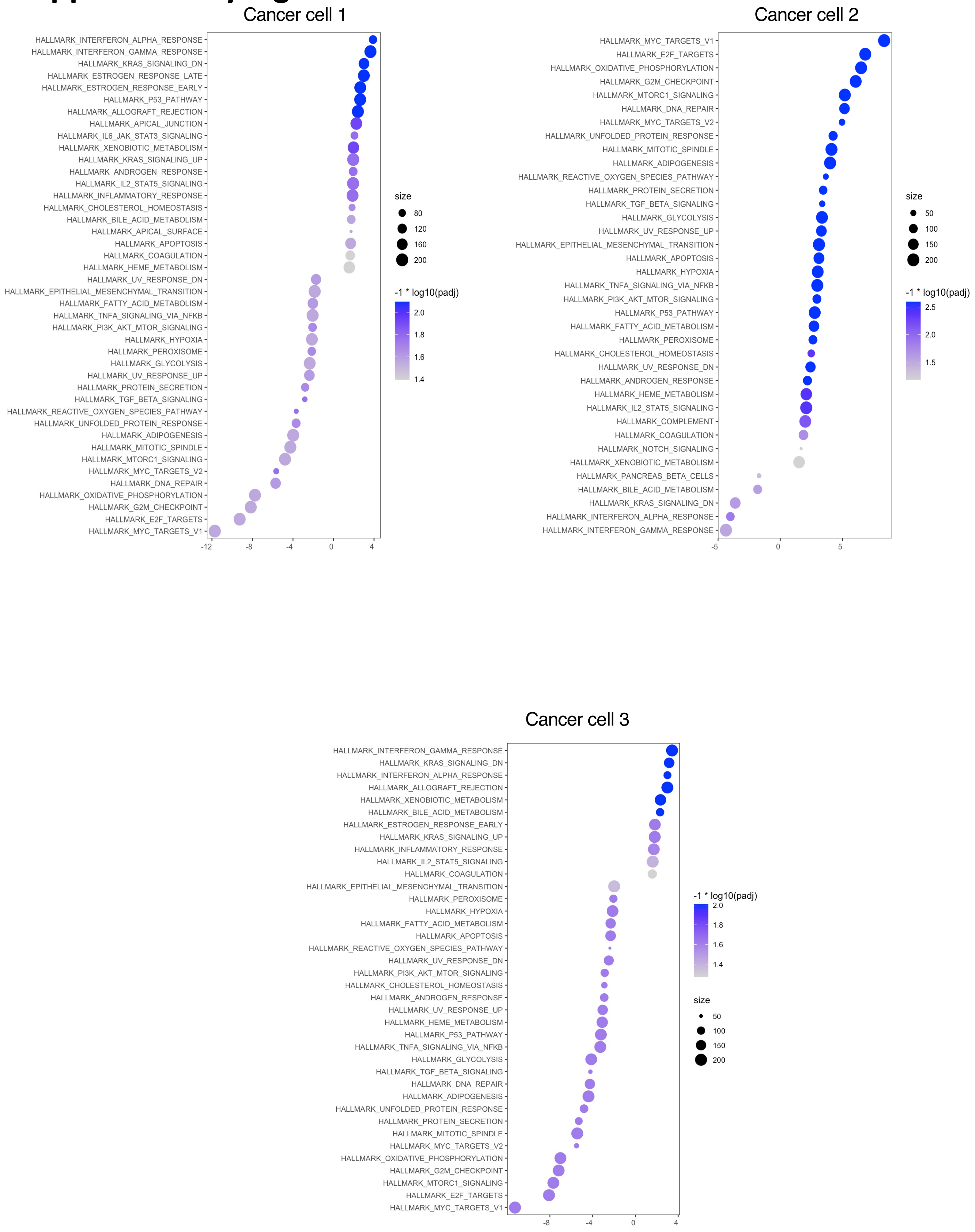

Supplementary Figure S3

A

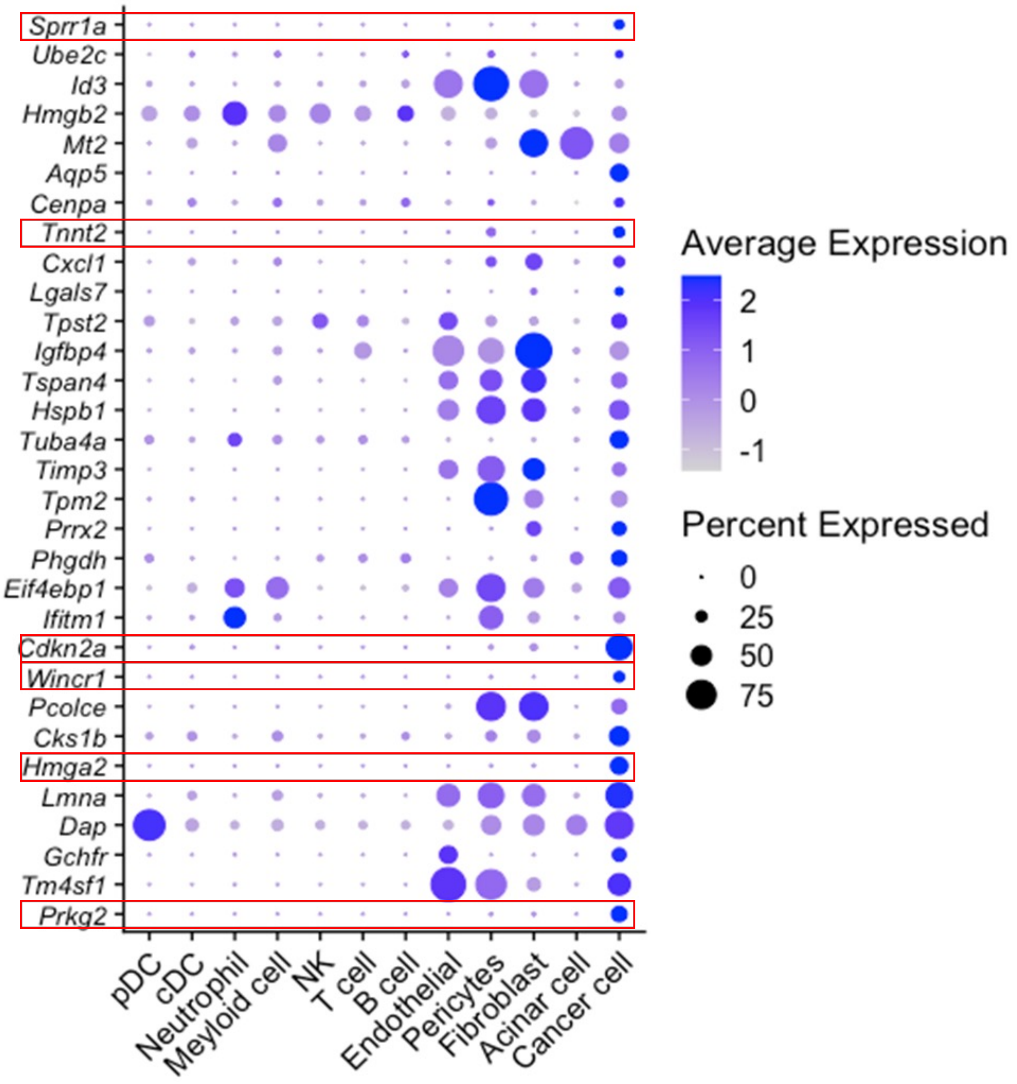

B

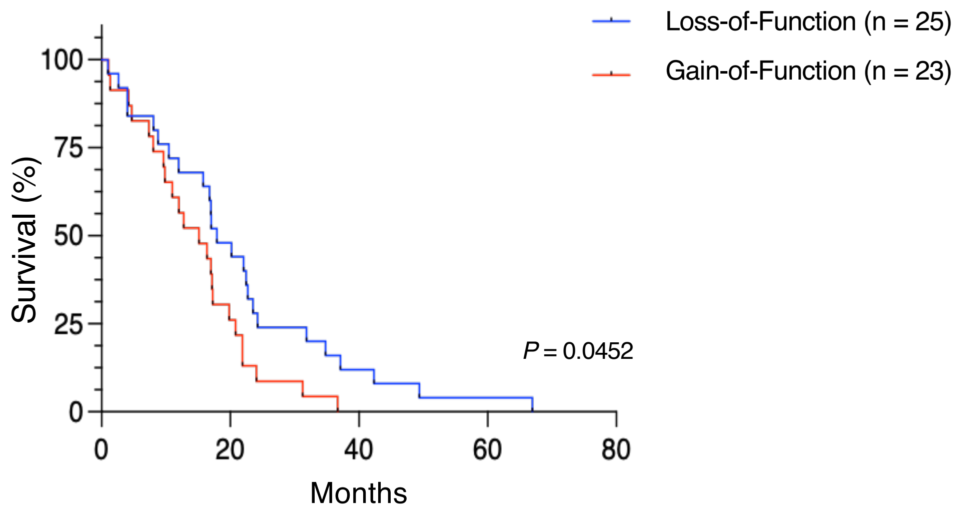

Supplementary Figure S4

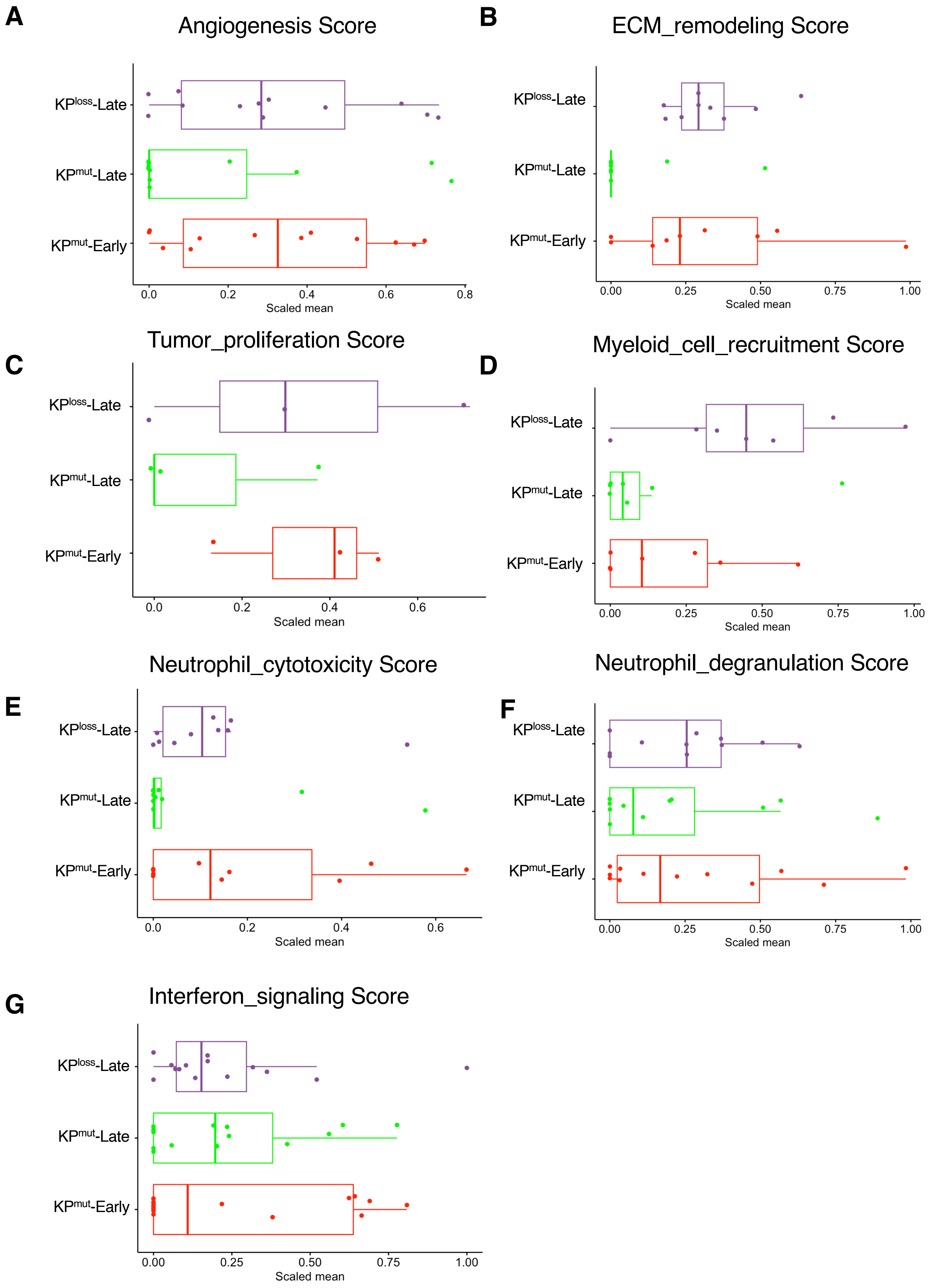

Supplementary Figure S5

A

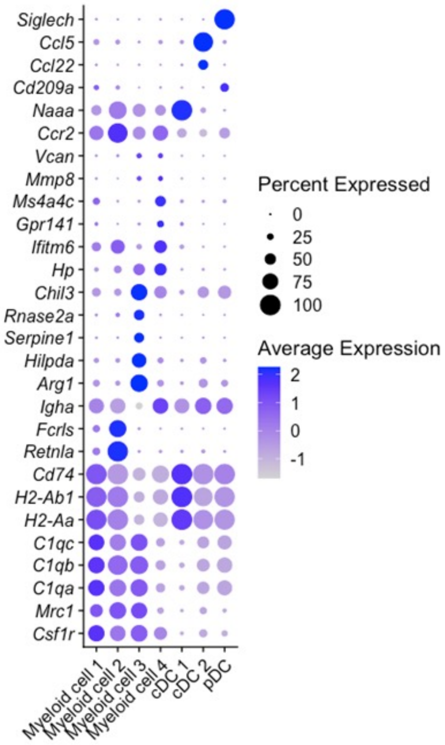

B

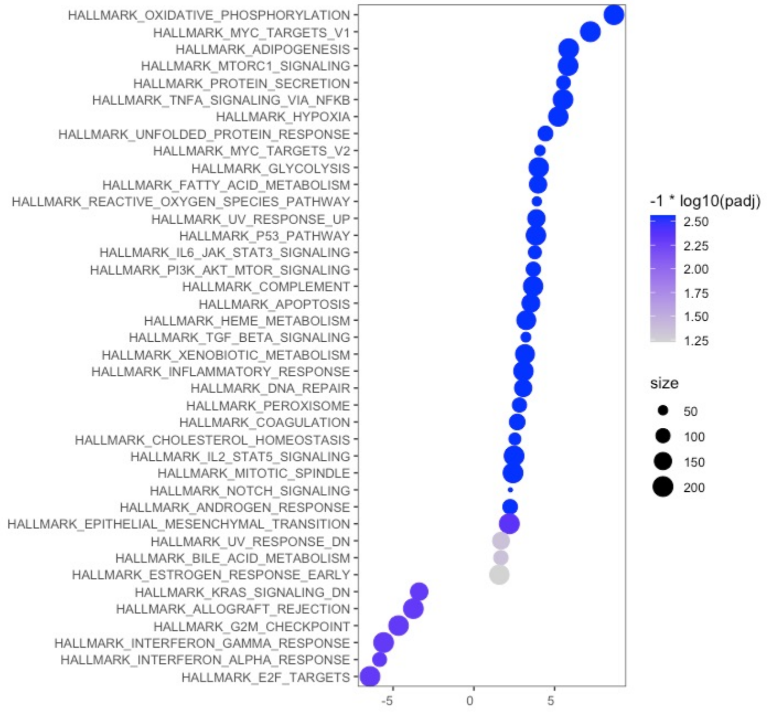

C

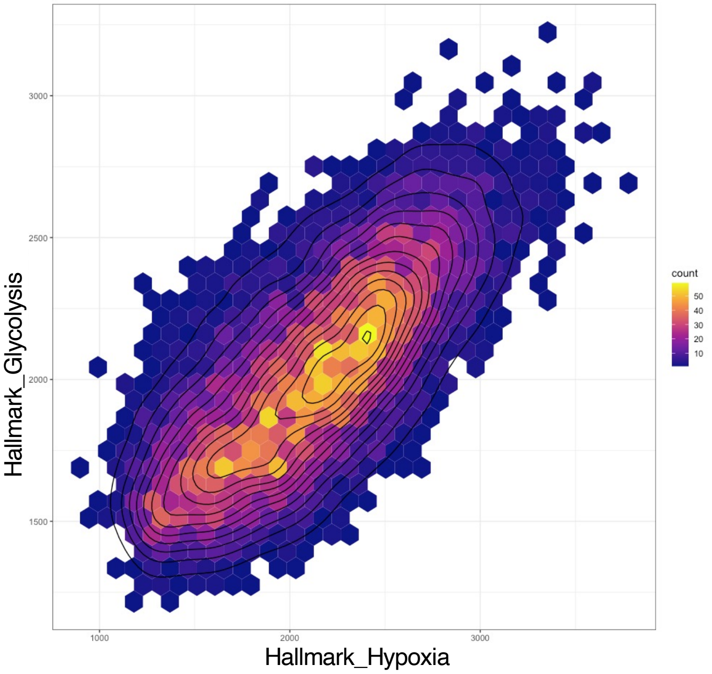

D

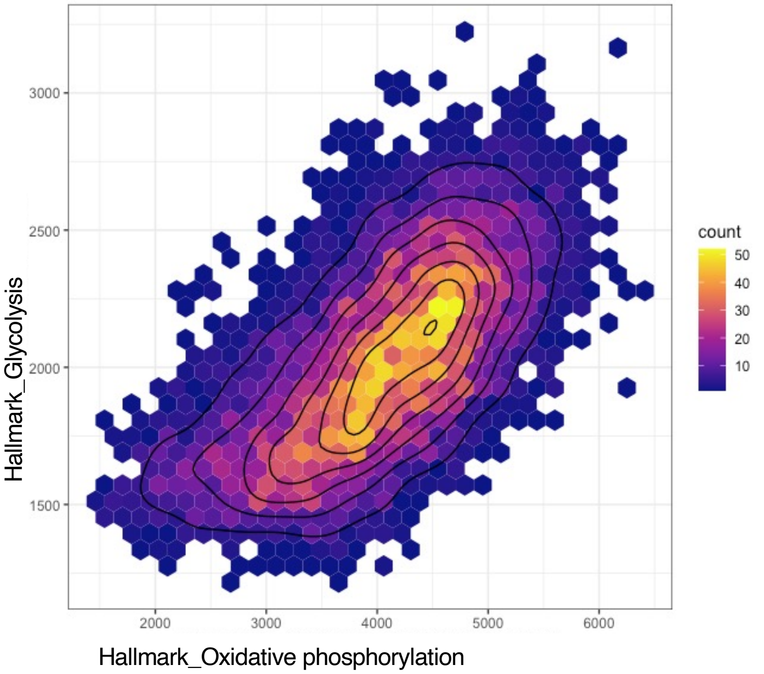

Supplement: Supplementary file 1 [file cells-12-02614-s001.zip › cells-2709541-supplementary.pdf]
